# Long-Term Effects of Maternal Diabetes on Blood Pressure and Renal Function in Rat Male Offspring

**DOI:** 10.1371/journal.pone.0088269

**Published:** 2014-02-05

**Authors:** Jie Yan, Xin Li, Rina Su, Kai Zhang, Huixia Yang

**Affiliations:** Department of Obstetrics and Gynecology, Peking University First Hospital, Beijing, China; Universidade Federal do Rio de Janeiro, Brazil

## Abstract

**Aims/Hypothesis:**

Gestational diabetes mellitus (GDM) is increasing rapidly worldwide. Previous animal models were established to study consequences of offspring after exposure to severe intrauterine hyperglycemia. In this study we are aiming to characterize the blood pressure levels and renal function of male offspring obtained from diabetic mothers with moderate hyperglycemia.

**Methods:**

We established a rat model with moderate hyperglycemia after pregnancy by a single intraperitoneal injection of streptozotocin (STZ). The male offspring were studied and fed with either normal diet or high salt diet after weaning. Arterial pressure and renal function were measured.

**Results:**

Arterial pressure of male offspring increased from 12 weeks by exposure to intrauterine moderate hyperglycemia. At 20 weeks, high salt diet accelerated the blood pressure on diabetic offspring compared to diabetic offspring fed with normal diet. We found offspring exposed to intrauterine moderate hyperglycemia had a trend to have a higher creatinine clearance rate and significant increase of urinary N-acetyl-β-D-glucosaminidase (NAG) excretion indicating an early stage of nephropathy progression.

**Conclusions/Interpretation:**

We observed the high blood pressure level and early renal dysfunction of male offspring obtained from diabetic mothers with moderate hyperglycemia. Furthermore, we investigated high salt diet after weaning on offspring exposed to intrauterine hyperglycemia could exacerbate the blood pressure and renal function. Renin angiotensin system (RAS) plays an important role in hypertension pathogenesis and altered gene expression of RAS components in offspring with *in utero* hyperglycemia exposure may account for the programmed hypertension. Therefore, our study provides evidence “fetal programming” of maternal diabetes is critical for metabolic disease development.

## Introduction

Gestational diabetes mellitus (GDM) is a common medical complication in pregnancy and has been rapidly increasing worldwide. GDM will bring health issues for both mothers and offspring, not only the early perinatal complications, but also the long-term consequences. Barker et al. initially brought up the concept of fetal programming that adult phenotype was affected by the intrauterine environment [1], [2]. Numerous studies focused on the relationship between adverse intrauterine environment and metabolic syndrome. Both epidemiologic investigations and animal studies have revealed long-term sequelae including cardiovascular abnormalities and metabolic syndrome in adult offspring exposed to intrauterine hyperglycemia [3]–[5]. Furthermore, an international multicenter Hyperglycemia and Adverse Pregnancy Outcomes (HAPO) study demonstrated that risk of adverse maternal, fetal, and neonatal outcomes continuously increased with maternal glucose levels, even within ranges thought to be normal for pregnancy [4]. Thus, it becomes challenging to focus on the adverse outcomes after exposure to intrauterine mild to moderate hyperglycemia.

Mechanisms involved in the fetal programming of hypertension are poorly understood. Little knowledge is available on the influence of intrauterine hyperglycemia, especially moderate hyperglycemia level. Animal studies provide the evidence that kidneys including the renin angiotensin system (RAS) may play an important role in hypertension programming [6]–[10]. In this study we established a GDM rat model of intrauterine moderate hyperglycemia induced by a single intraperitoneal injection of streptozotocin (STZ). We characterized the blood pressure levels and renal function of male offspring obtained from diabetic mothers. Furthermore, diseases are partially influenced by life style. Here, we investigated the possible effects of high salt diet on offspring exposed to intrauterine hyperglycemia.

## Materials and Methods

### Ethics Statement

All animal protocols were reviewed and approved by the Institutional Animal Care and Use Committee of Peking University First Hospital (J201010). All surgery was performed under Chloral hydrate anesthesia, and all efforts were made to minimize suffering.

### Animals and tissue isolation

**Figure 1:**
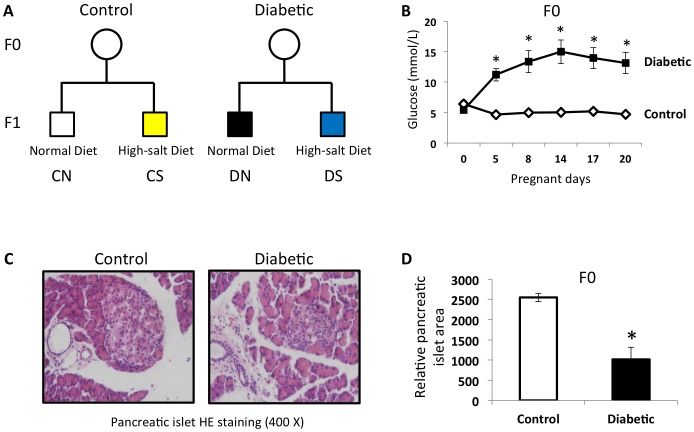
GDM rat model with moderate hyperglycemia was induced by streptozotocin. (**A**) Study design: The male offspring were studied and fed with either normal diet or high salt diet (8% NaCl) after weaning: control group fed with normal diet (CN, n = 9) or high salt diet (CS, n = 9), diabetic group fed with normal diet (DN, n = 9) or high salt diet (DS, n = 9). (**B**) GDM rat model with moderate hyperglycemia after pregnancy was established by a single intraperitoneal injection of streptozotocin (STZ). The average glucose level of diabetic mothers during pregnancy was 11.2–15.0 mmol/L. (**C**) HE staining images (400X) of pancreatic islets obtained from control and diabetic rat mothers. (**D**) Pancreatic islets area and diameters were measured using an image analysis program, Image-Pro Plus 6.0, Media Cybernetics. Ten different fields were analyzed for each group. Results are mean ± sem. * indicates p<0.05.

Room temperature and humidity were maintained stable and under a 12-hour light/dark cycle. At the age of 12 weeks, female Wistar rats (Vital River Laboratory Animal Technology Co., Ltd., Beijing, China) were mated with normal males. Onset of pregnancy was determined by the presence of a copulation plug after overnight mating (designated as day 0 [D0] of pregnancy). After a 12-h fasting, the female rats were randomly divided into two groups: control group (Control), intrauterine hyperglycemia group with GDM (Diabetic). Rats in Diabetic group were injected with a single intraperitoneal injection of streptozotocin (STZ, Sigma, Beijing, China) in citrate buffer (pH 4.4) at a dose of 25 mg/kg. Control pregnant female rats received an equal volume of citrate buffer. On D5 of pregnancy and every three days afterwards, diabetes was confirmed by measuring blood glucose concentration via the tail vein. The pregnant rats were allowed to deliver spontaneously. The male offspring were studied and fed with either normal diet or high salt diet (8% NaCl) after weaning: Control group fed with normal diet (CN, n = 9) or high salt diet (CS, n = 9), Diabetic group fed with normal diet (DN, n = 9) or high salt diet (DS, n = 9) (Fig. 1A). The phenotypes of male offspring were characterized and were killed at 26 weeks. Blood, heart, liver, pancreas, kidney and fat pads were carefully dissected. Tissues were dissected from visible blood vessels and immediately frozen in liquid nitrogen.

### Oral glucose tolerance test

Rats were fasted overnight and given glucose (2 g/kg) for the oral glucose tolerance test (OGTT). Blood samples were collected from the tail vein before (time  = 0) and after 15, 30, 60 and 120 min of glucose administration given by gavage.

### Measurement of blood pressure

Blood pressure and heart rate were determined in conscious rats from all groups by an indirect tail-cuff method (Indirect blood pressure meter BP-98A, Softron™, Tokyo, Japan). Before the measurements, rats were placed in the restrainers for several times and the measures were always performed by the same person. The pressure records were made after a 15 to 20 min of quietude. Three stable consecutive measurements of blood pressure and heart rate were averaged.

### Metabolic cages

All male offspring were housed separately for 24 hours (at 14, 18, 22, 24 weeks) in metabolic cages (Peking University Health Science Center Experimental Animal Unit) with free access to food (normal diet or high salt diet) and water. Food intake, water consumption and urinary volume were measured. Urine creatinine concentration, sodium, potassium, chlorine excretion and N-acetyl-β-D-glucosaminidase (NAG) were also measured. Creatinine clearance rate (Ccr) was calculated by the formula: Ccr (ml/min)  =  urine creatinine concentration (µmol/L) X urine volume (ml) in 24 hours/serum creatinine concentration (µmol/L)/1440.

### Morphometric study

The morphologic evaluation was obtained by the following methodology: pancreases and kidneys were dissected out rapidly, cleaned of connective tissue, weighed and fixed. Pancreases and kidneys were wax embedded and histologic sections (5 µm width) were performed. Sections cut were stained with hematoxilin and eosin to morphologic analysis. Pancreatic islet or glomerular area and diameters were measured using an image analysis program, Image-Pro Plus 6.0, Media Cybernetics. Ten different fields were analyzed for each group. The number of glomeruli was also calculated in these same fields.

### Nucleic acid purification and Real Time PCR

For RNA extraction, kidney samples were homogenized in 1 ml of Trizol reagent and RNA was purified according to recommendations of the manufacturer. RNA was used as a template for cDNA synthesis using First Strand Synthesis Kit (Fermentas, USA) and cDNA quantity was measured using real time PCR with the ABI PRISM 7300 sequence detector system and fluorescence-based SYBR-green technology. PCR was performed in a final volume of 20 µl, consisting of diluted cDNA sample, 1× SYBR-green PCR Master Mix (Molecular Probes, USA), primers optimized for each target gene and nuclease-free water. All samples were analyzed in duplicates. Primers were designed using Primer Premier 5.0 software. The following primers were used, *Angiotensinogen*: sense 5′ AGC ATC CTC CTT GAA CTC CA 3′, antisense 5′ TCT CAC CCC AGT GTC CAA AC 3′; *Renin*: sense 5′ TGT GCA TAC TGG CTC TCC AA 3′, antisense 5′ AAA GCA GGG AAG GGT GAG TG 3′; *Angiotensin-converting enzyme 1*: sense 5′ TGG AAC GAA TAC GCA GAG 3′, antisense 5′ AGC AAA CAT TGG CTA CAC T 3′; *Angiotensin II type 1A receptor*: sense 5′ AGA GTC AGG AGC TGG ATG GA 3′, antisense 5′ GAT GTG TGG ACT TGG GTA ACA 3′; *Angiotensin II type 1B receptor*: sense 5′ TTT GGG CTA AGC AGC TCA CT 3′, antisense 5′ AGC AGT TTG GCT TTG CAA CT 3′; *Beta-actin*: sense 5′ AGC CAT GTA CGT AGC CAT CC 3′, antisense 5′ GCT GTG GTG GTG AAG CTG TA 3′.

### Western blot analysis

Kidney biopsies were homogenized in RIPA Lysis buffer (KeyGEN BioTECH, KGP702; Nanjing, China) and 1 mM PMSF (amresco, M221; USA). Protein was determined by the BCA (bicinchoninic acid) protein assays kit from KeyGEN BioTECH (Nanjing, China). Samples were resuspended in Laemmli buffer, and proteins were separated on 10% SDS-PAGE. Proteins were transferred to polyvinylidenedifluoride membranes (APPLYGEN, P2110; Beijing, China) and subjected to western blot analysis. After incubation with primary antibody, membranes were washed and incubated with secondary antibody linked to horseradish peroxidase (ZSGB-BIO; Beijing, China). Multiple exposures were used to ensure that proteins were detected in linear range of protein band saturation. Seven samples from each group were loaded in two separate gels and they were treated equally during gel electrophoresis, transfer, blocking, antibody incubation as well as detection. Results were quantified by densitometry using AlphaEaseFC, FluorChem SA for Windows (Alpha Innotech Corporation; California, USA). The following antibodies were used for the western blot analysis: ANGIOTENSINOGEN (EPITOMICS, Cat.#3208-1; California, USA), RENIN (Sigma, Cat.#QC12090; Saint Louis, USA), ANGIOTENSIN-CONVERTING ENZYME 1 (abcam, Cat.#ab77990; Cambridge, USA), ANGIOTENSIN II TYPE 1 RECEPTOR (EPITOMICS, Cat.#5172-1; California, USA) and GAPDH (TA-08) were purchased from ZSGB-BIO (Beijing, China).

### Statistics

One-way ANOVA was used to determine the comparisons among four groups. Post-hoc comparisons using Tukey's test were performed when a significant F-score were detected. Comparisons between two groups were performed using two-tailed unpaired Student's *t* test. All values are presented as mean ± SEM. Statistically significant differences were defined as P<0.05.

## Results

### Intrauterine moderate hyperglycemia induced high birth weight and glucose intolerance in male offspring

We established a GDM rat model with moderate hyperglycemia after pregnancy by a single intraperitoneal injection of streptozotocin (STZ). The average glucose level of diabetic mothers during pregnancy was 11.2–15.0 mmol/L (Fig. 1B). Further morphological study of pancreases confirmed the smaller pancreatic islets in diabetic mothers (Fig. 1C, 1D).

**Figure 2:**
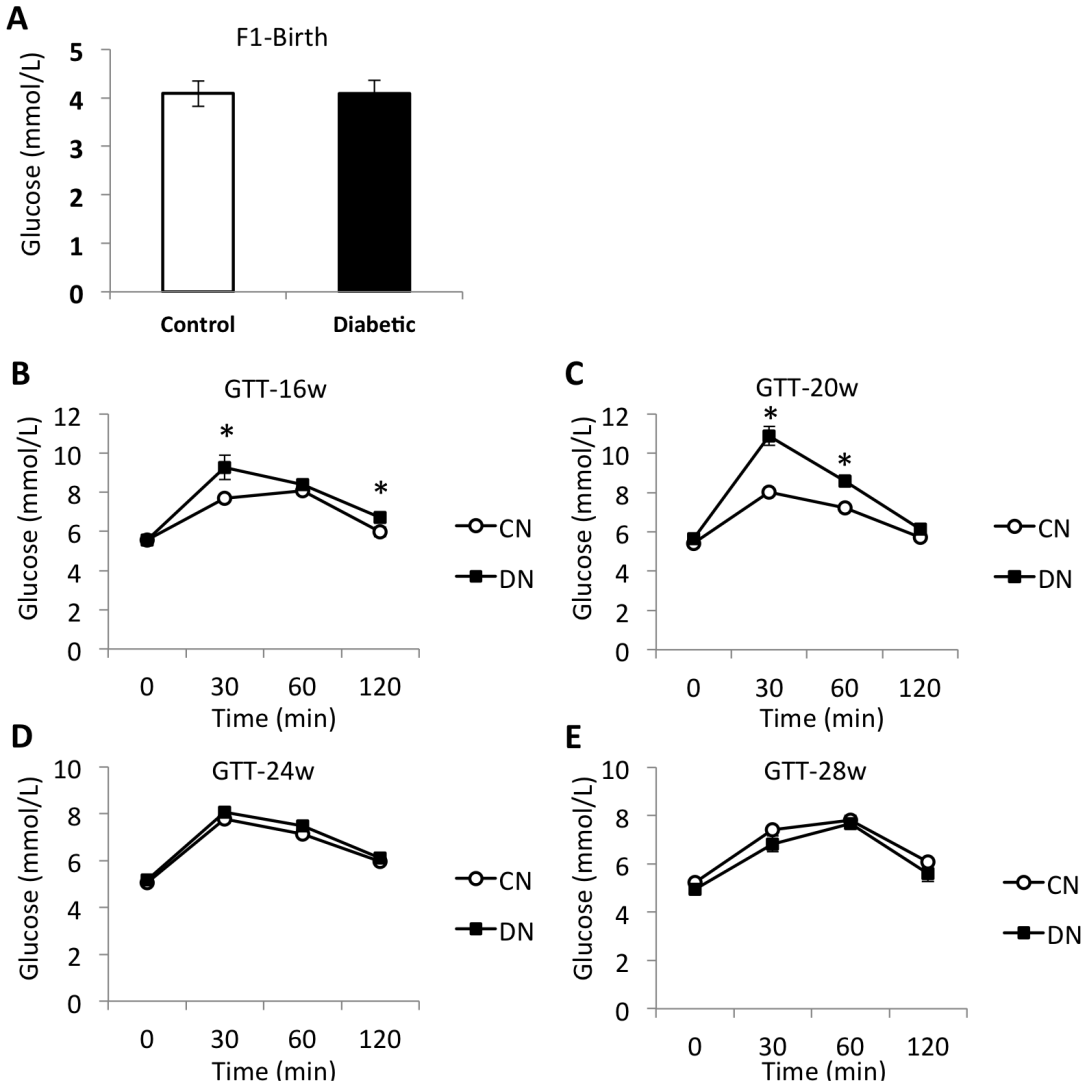
GDM rat model induced glucose intolerance in male offspring. (**A**) No significant difference of blood glucose level at birth in offspring born from control and diabetic mothers. (**B–E**) Glucose tolerance test (GTT) was performed at 16, 20, 24, 28 weeks using glucose (2 g/kg body weight) given by gavage. N = 9. Results are mean ± sem. * indicates p<0.05.

There was no significant difference of blood glucose level at birth in offspring born from control and diabetic mothers (Fig. 2A). We further performed glucose tolerance test (GTT) using glucose (2 g/kg body weight) given by gavage. At 16 weeks, impaired glucose tolerance (IGT) was found in diabetic offspring, whose blood glucose level significantly increased at 30 min and 120 min after glucose load (Fig. 2B). At 20 weeks, IGT was also found at 30 min and 60 min in glucose tolerance test (Fig. 2C). We did not find any difference at 24 and 28 weeks.

The birth weight of male offspring obtained from diabetic mothers was significantly higher than the controls (Table 1). However, there was no significant difference after 3 weeks. The body weight of offspring fed with high salt diet was lower than offspring fed with normal diet (CS vs. CN, DS vs. DN) (Table 1).

**Table 1:** Body weight of male offspring.

	n	At birth (gram)	3 weeks (gram)	7 weeks (gram)	11 weeks (gram)	15 weeks (gram)	19 weeks (gram)	23 weeks (gram)
**CN**	9	6.55±0.15	35.59±1.46	216.67±6.87	393.89±11.23	458.56±14.95	519.56±16.56	561.89±18.76
**DN**	9	7.38±0.20^a^	44.09±1.97^a^	232.67±8.54	400.89±13.35	487.89±15.61	534.33±16.96	574.89±21.32
**CS**	9	6.72±0.15	37.54±2.16	229.22±5.38	391.11±10.75	455.67±10.28	489.22±14.49	514.67±12.00^a^
**DS**	9	7.54±0.14^b^	39.22±2.81	222.22±7.55	369.22±9.86	443.67±12.14^c^	486.00±14.79^c^	522.89±14.25^c^

Data are means ± sem.

a p<0.05 vs. CN;

b p<0.05 vs. CS;

c p<0.05 vs. DN.

### Intrauterine moderate hyperglycemia induced high blood pressure in male offspring

**Figure 3:**
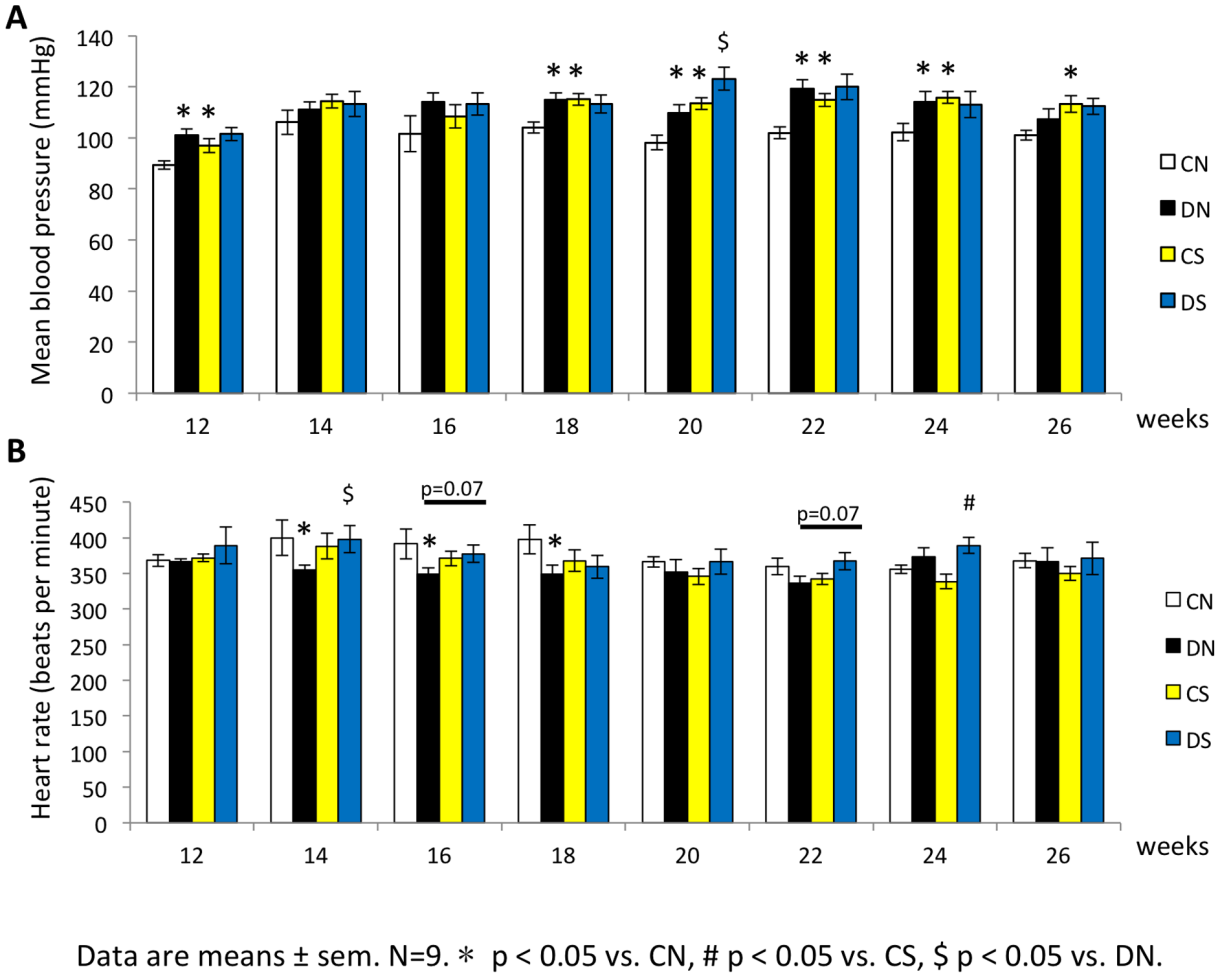
GDM rat model induced high blood pressure in male offspring. (**A–B**) Blood pressure and heart rate were determined in conscious rats from all offspring groups by an indirect tail-cuff method. Three stable consecutive measurements of blood pressure and heart rate were averaged. Data are means ± sem. N = 9. * p<0.05 vs. CN, # p<0.05 vs. CS, $ p<0.05 vs. DN.

Mean arterial pressure of male offspring was increased from 12 weeks by exposure to intrauterine moderate hyperglycemia. High salt diet also induced hypertension on control offspring. At 20 weeks, high salt diet could accelerate the blood pressure change on diabetic offspring compared to control diet (Fig. 3A). Heart rate of diabetic male offspring decreased at 14 weeks to 18 weeks. However, heart rate did not differ by high salt diet (Fig. 3B).

### Effect of intrauterine moderate hyperglycemia on renal function

**Figure 4:**
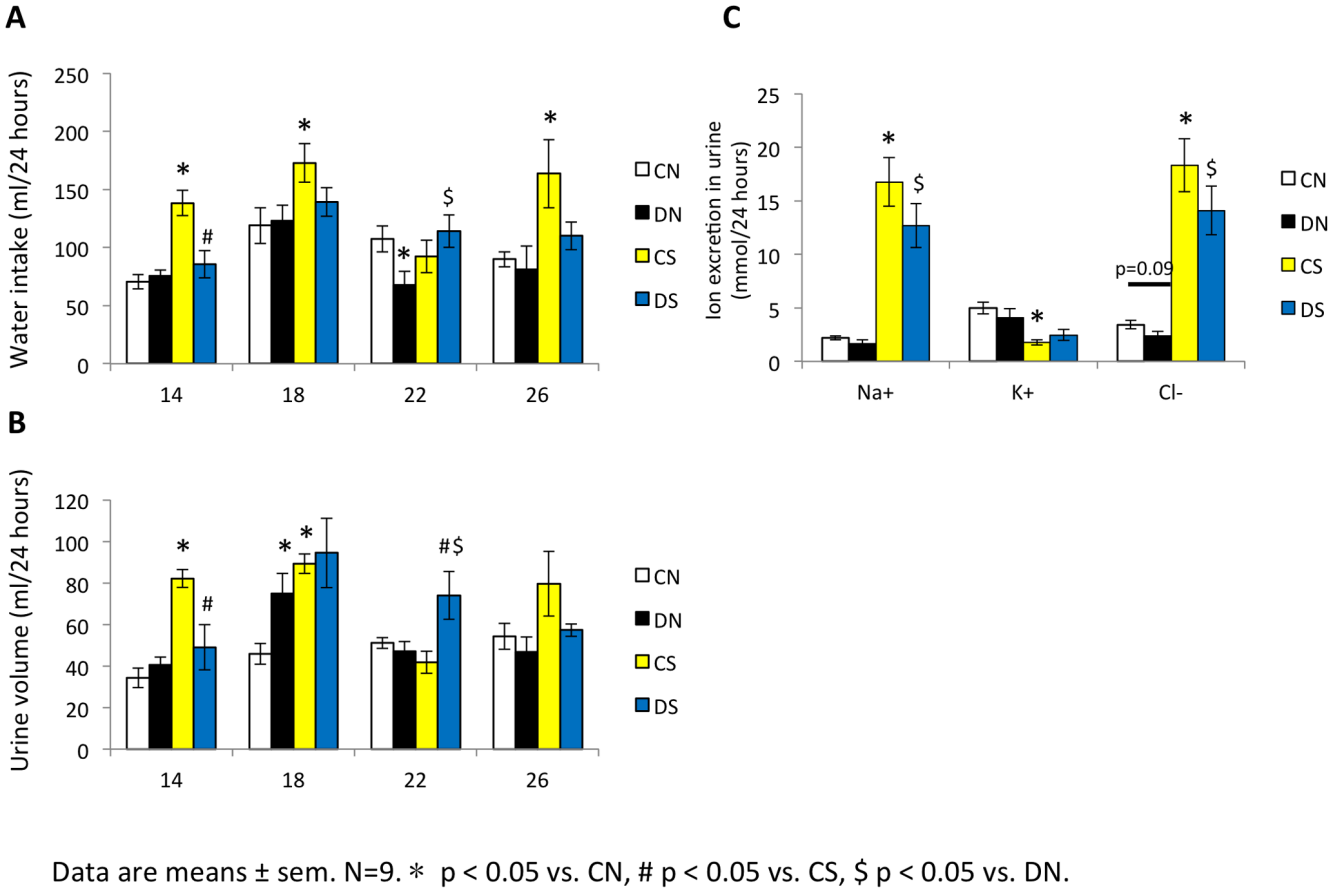
Water intake, urine volume and urinary ion excretion in male offspring. *In vivo* studies were carried out in metabolic cages with rats to compare water intake (**A**), urine volume (**B**) and Na^+^, K^+^, Cl^−^ excretion (**C**) between offspring groups. Data are means ± sem. N = 9. * p<0.05 vs. CN, # p<0.05 vs. CS, $ p<0.05 vs. DN.

Diabetic offspring group had a higher kidney weight than control offspring (DN vs. CN, p<0.05) (Table 2). The percentage of kidney weight/body weight in groups fed with high salt diet was higher than groups fed with normal diet (CS vs. CN, DS vs. DN, p<0.05) (Table 2). However, the number of glomeruli did not differ in male offspring groups (data not shown). *In vivo* studies were carried out in metabolic cages with rats to compare creatinine clearance rate (Ccr) and Na^+^, K^+^, Cl^−^ excretion between offspring groups. Na^+^, K^+^, Cl^−^ excretion was similar between diabetic offspring and control offspring at 26 weeks (DN vs. CN, p>0.05). The DN group had a higher urine creatinine (Ucr) level and a trend to have a higher Ccr value (2.01±0.32 vs. 1.34±0.11, p = 0.07) (Table 3). By contrast, CS and DS group excreted more Na^+^, Cl^−^ and less K^+^ per day compared to the CN and DN group (Fig. 4C). It was obvious CS group had more water intake and more urine excretion on average compared to CN group (Fig. 4A, 4B) whereas the Ucr and Ccr values were significantly lower than the CN group (Table 3). It was also revealed impaired Ccr in DS group compared to DN group (Table 3). It should be noted that the serum creatinine levels in all groups were similar. Nevertheless, we investigated the urinary N-acetyl-β-D-glucosaminidase (NAG) excretion, a biomarker that can detect earlier stage of tubular cell disturbances, was significantly increased in DN and CS groups compared to CN group. DS group had a trend to excrete more NAG than CS group (p = 0.08) (Table 3).

**Table 2:** Kidney weight of male offspring.

	n	Left kidney weight (gram)	Right kidney weight (gram)	Average kidney weight (gram)	Average kidney weight/body weight (%)
**CN**	9	1.71±0.05	1.83±0.08	1.77±0.06	0.28
**DN**	9	1.97±0.10^a^	2.06±0.09	2.02±0.09^a^	0.33^a^
**CS**	9	1.93±0.10^a^	2.15±0.10^a^	2.04±0.09^a^	0.36^a^
**DS**	9	2.01±0.10	2.06±0.11	2.03±0.09	0.38^c^

Data are means ± sem.

a p<0.05 vs. CN;

c p<0.05 vs. DN.

**Table 3:** Renal function of male offspring at 26 weeks.

		Scr (µmol/L)	Ucr (mmol/L)	Ccr (ml/min)	NAG (U/L)
**CN**	9	55.94±0.80	2.16±0.28	1.34±0.11	6.71±0.40
**DN**	9	54.24±1.00	3.64±0.64^a^	2.01±0.32	12.33±2.53^a^
**CS**	9	55.78±0.67	1.24±0.17^a^	1.03±0.10^a^	12.00±1.29^a^
**DS**	9	54.68±0.95	1.63±0.16^c^	1.17±0.12^c^	15.56±1.56

Serum creatinine (Scr), urine creatinine (Ucr) and N-acetyl-β-D-glucosaminidase (NAG) were measured. Creatinine clearance rate (Ccr) was calculated by the formula: Ccr (ml/min)  =  urine creatinine concentration (µmol/L) X urine volume (ml) in 24 hours/serum creatinine concentration (µmol/L)/1440. Data are means ± sem.

a p<0.05 vs. CN;

c p<0.05 vs. DN.

### Intrauterine moderate hyperglycemia affected gene expression of RAS components

**Figure 5:**
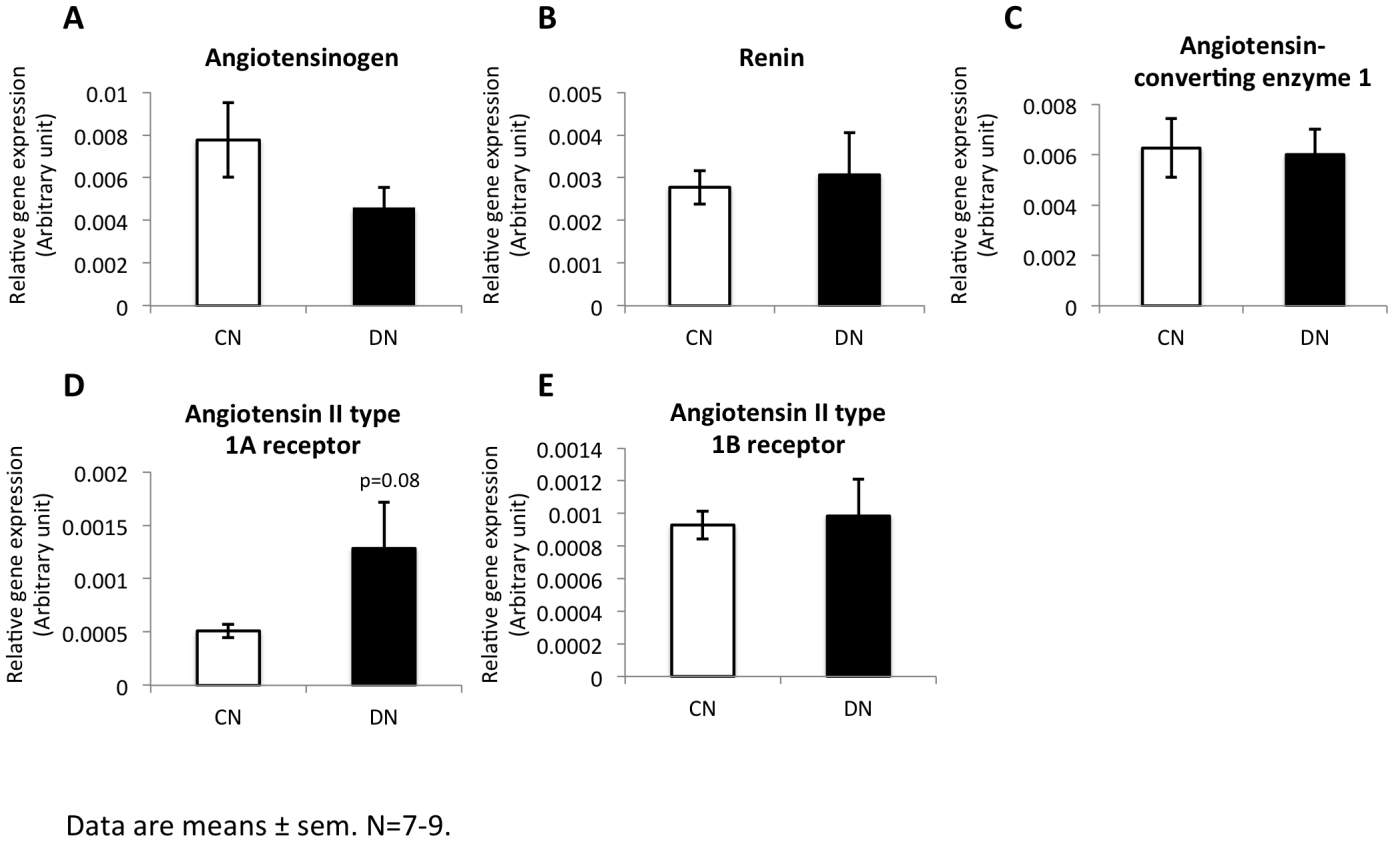
Intrarenal expression of RAS components. Components of RAS in kidney in offspring groups were measured by real-time quantitative PCR. The gene expression of angiotensinogen, renin, angiotensin converting enzyme 1, angiotensin II type 1A receptor and angiotensin II type 1B receptor (**A–E**) was detected.

**Figure 6:**
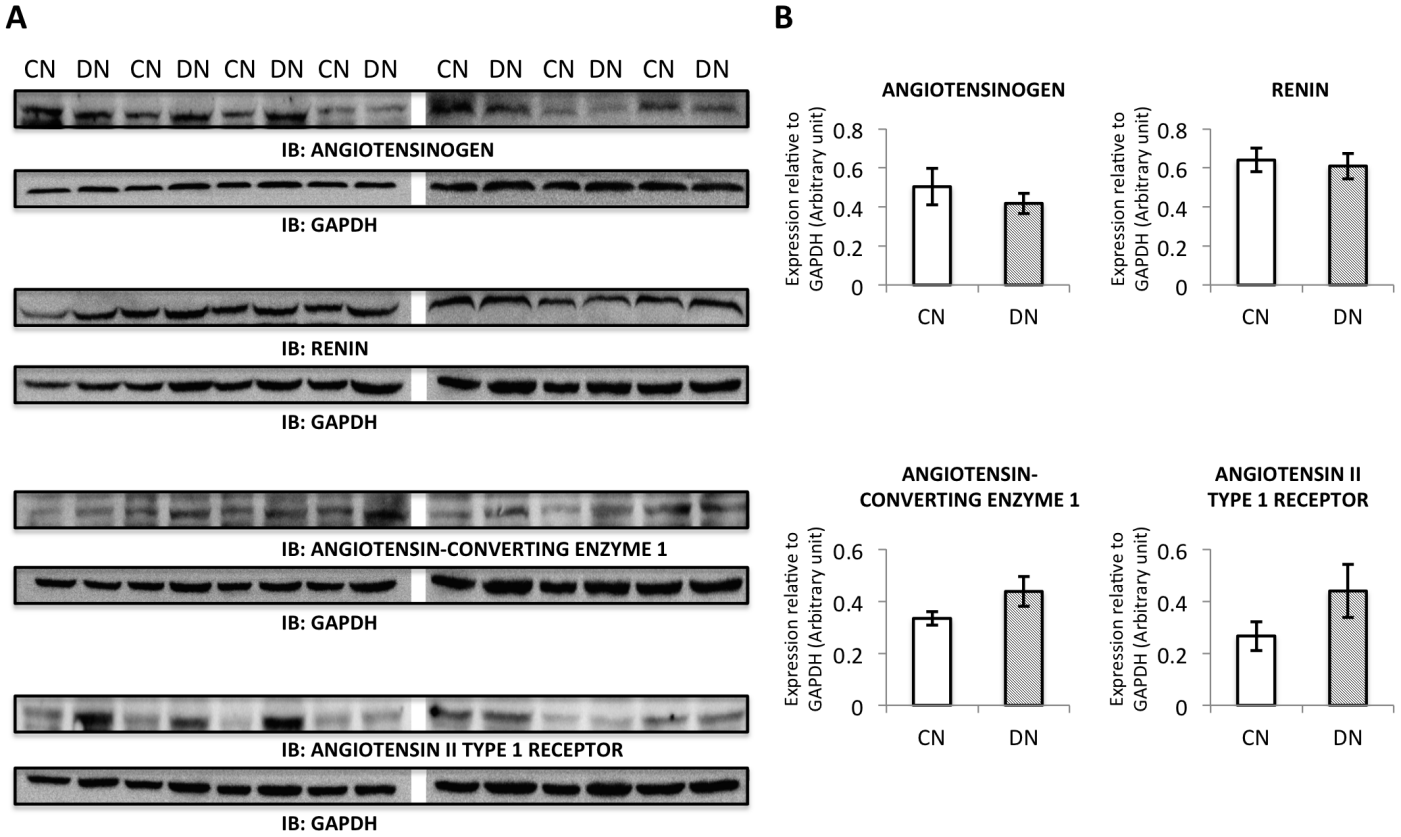
Protein expression of RAS components. Components of RAS in kidney in offspring groups were measured by western blot analysis. The protein expression of ANGIOTENSINOGEN, RENIN, ANGIOTENSIN CONVERTING ENZYME 1 and ANGIOTENSIN II TYPE 1 RECEPTOR was detected. Seven samples from each group were loaded in two separate gels and they were treated equally during gel electrophoresis, transfer, blocking, antibody incubation as well as detection.

RAS plays an important role in the regulation of body fluid balance and tubular transport function. We measured various components of RAS in kidney in offspring groups by real-time quantitative PCR. The expression of *angiotensin II type 1A receptor* had a trend to increase in DN group compared to the control (Fig. 5D). There was no change for *angiotensinogen*, *renin*, *angiotensin converting enzyme 1* and *angiotensin II type 1B receptor* (Fig. 5A, 5B, 5C, 5E). The protein expression levels of RAS components were not altered measured by western blot analysis (Fig. 6A, 6B).

## Discussion

The study of the Pima Indians initially provided the evidence that maternal hyperglycemia could lead to adult disease in offspring [11]. The epidemiologic studies demonstrated the Pima Indian population in Arizona revealed the highest prevalence of Type 2 diabetes mellitus among children and adults. The offspring of diabetic mothers also have more chances to get obesity, hypertension and dyslipidemia [12]–[14]. Animal models have been established to study long-term sequelae including cardiovascular abnormalities [15], [16] and metabolic syndrome in adult offspring exposed to intrauterine hyperglycemia. The mechanisms underlying are still under discussion. HAPO study recently reported the risk of adverse maternal, fetal, and neonatal outcomes with maternal mild hyperglycemia, even within ranges previously thought to be normal for pregnancy [4]. The incidence of GDM in China is as high as 17.5% [17]. Actually, most of the GDM women with high glucose levels have been well controlled. Thus, it is more realistic to study effects of intrauterine mild to moderate hyperglycemia exposure.

Previous GDM animal models treated with streptozotocin mostly developed hyperglycemia with glucose levels more than 15 mmol/L [16], [18]. In our study, we established a GDM rat model with glucose level of 11.2–15.0 mmol/L by a single intraperitoneal injection of streptozotocin at a lower dose of 25 mg/kg. Interestingly, offspring of diabetic mothers have higher birth weight, which can be used as a model of human macrosomia born from GDM mothers. Offspring of diabetic mothers developed impaired glucose tolerance at 16 and 20 weeks and hypertension after exposure to intrauterine moderate hyperglycemia. It is indicating that hypertension found in the adult life may already be programmed *in utero*.

There is a large body of evidence showing a correlation of dietary salt intake with progression of hypertension [19]. Furthermore, individuals responded differently to an increased dietary salt intake. Although the reasons for salt induced hypertension are heterogeneous, it is mainly due to renal dysfunction to excrete sodium. We further fed offspring with high salt diet to investigate if offspring exposed to intrauterine hyperglycemia are salt sensitivity or not. We found DS group is prone to develop hypertension given salt diet at 20 weeks. In other words, salt loading could exaggerate the programmed hypertension in certain period in offspring that have been exposed to intrauterine hyperglycemia.

Next, we examined the renal function in offspring. Elevation in the glomerular filtration rate (GFR) and glomerular hypertrophy occur early in the course of Type 2 diabetes mellitus [20], [21] and increased renal size accompanies the rise in GFR. Similarly, we found offspring exposed to intrauterine hyperglycemia had a trend to have a higher GFR assessed by creatinine clearance rate indicating an early stage of nephropathy progression. Besides, we also found DN group had larger kidneys compared to CN group. Further morphologic study of kidney showed focal segmental glomerulosclerosis changes in offspring exposed to intrauterine hyperglycemia (data not shown). N-acetyl-β-D-glucosaminidase (NAG), is a lysosomal enzyme with high molecular-weight found in various tissues. In normal conditions, it cannot pass and leak into glomerular ultrafiltrate due to the high molecular-weight. The urinary level of this enzyme changes reflect the proximal tubular cell necrosis degree. It can be used as a biomarker showing earlier stage of tubular cell disturbances. More urinary NAG excretion was found in offspring born from diabetic mother. A change with increased NAG leakage (CS vs. CN, p<0.05) and decreased Ccr (CS vs. CN, p<0.05) also occurred in high salt diet group. Salt loading could accelerate the impaired renal function (Ccr) induced by intrauterine hyperglycemia exposure (DS vs. DN, p<0.05).

The renin-angiotensin system (RAS) is an important regulator of blood pressure, body fluid balance as well as electrolyte balance. In the kidney, all the components of RAS are expressed locally during nephrogenesis and RAS plays an important role of normal morphological development of the kidney [22]. The current study showed in offspring exposed to intrauterine hyperglycemia compared to controls, an increase trend of *angiotensin II type 1A receptor* mRNA expression in RAS pathway in the kidney. It has been reported 20% of the total nephron number is present at birth [23]. Hence, *in utero* exposure to hyperglycemia may influence renal function through RAS function.

Here, we cannot explain the increasing blood pressure in offspring with *in utero* hyperglycemia exposure by reduced nephron number and decreased GFR, since we investigated neither nephron number difference nor GFR decrease between DN and CN. Nevertheless, the compensatory mechanism of renal function with impaired nephrogenesis may be proposed. The hypertension we observed in offspring born from diabetic mother may involve several mechanisms. Although the mechanisms are still under investigation, ‘fetal programming’ may contribute to regulate blood pressure and affect renal function in offspring exposed to intrauterine hyperglycemia.

In conclusion, our study suggests that offspring exposed to intrauterine moderate hyperglycemia could develop glucose intolerance, hypertension and early stage of renal impairment. It is indicating that ‘fetal programming’ may contribute to disease progression in adult. RAS may involve in renal development in this process. We also provide the evidence that high salt diet may accelerate hypertension and renal dysfunction in offspring exposed to intrauterine hyperglycemia. It implies that offspring exposed to *in utero* insults may determine the disease in adult and salt loading after birth may exaggerate the disease development.

The concept of ‘DOHaD’ (Developmental Origins of Health and Disease) has shed light on the molecular pathogenesis of human diseases. The underlying mechanism is still obscure and epigenetic mechanisms may contribute to explain the phenomenon. Changes in epigenetic mechanism may influence the pathogenesis and progression of the metabolic disease in humans at an early stage. Further studies should be focused on epigenetic modifications of *in utero* exposure.
